# Supplementary material for: Preservation of three-dimensional anatomy in phosphatized fossil arthropods enriches evolutionary inference
Source: eLife. 2016 Feb 8;5:e12129. doi: 10.7554/eLife.12129 (PMC4758943; doi:10.7554/eLife.12129)
Supplement: Supplementary file 1. — Click on the figure to start interactive 3D view; switch between views by using the menu (Adobe Reader 8.1 or higher required). DOI: http://dx.doi.org/10.7554/eLife.12129.010 [file elife-12129-supp1.zip › schwermann_et_al_supplementary_file1.pdf]

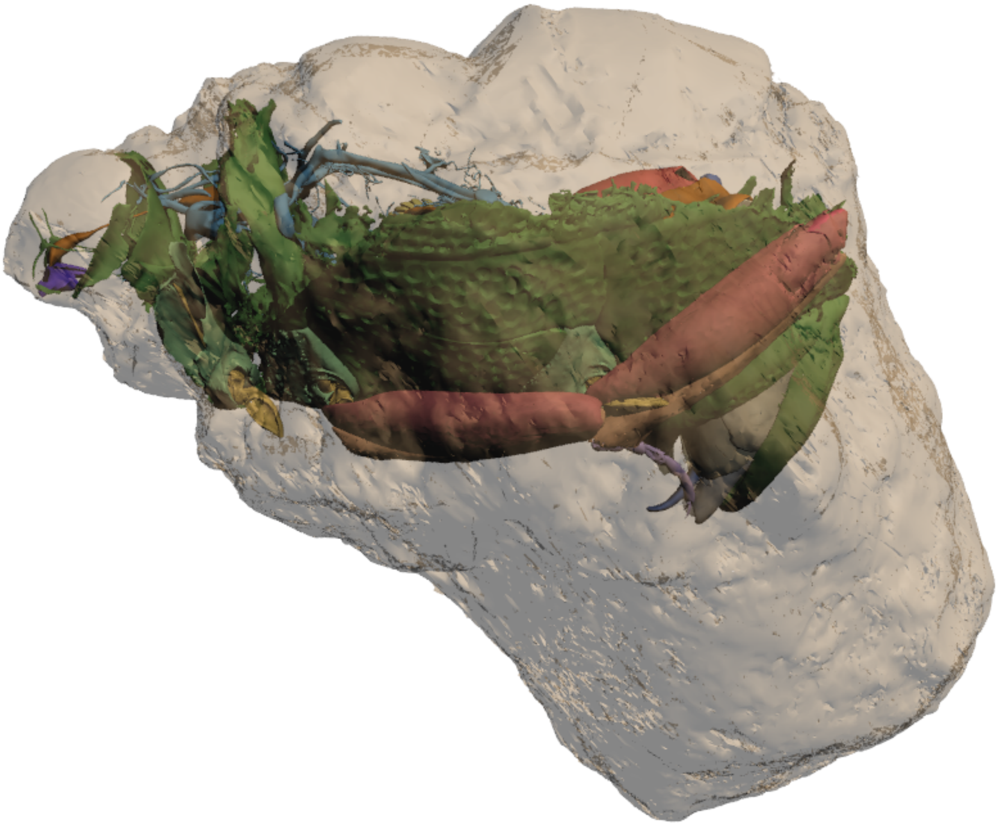

Supplementary File 1. Interactive 3D reconstruction of *Onthophilus intermedius* specimen F1994. Click on the figure to start interactive 3D view; switch between views by using the menu (Adobe Reader 8.1 or higher required).
